# Supplementary material for: Oncolytic virus-derived type I interferon restricts CAR T cell therapy
Source: Nat Commun. 2020 Jun 24;11:3187. doi: 10.1038/s41467-020-17011-z (PMC7314766; doi:10.1038/s41467-020-17011-z)
Supplement: Supplementary file 1 — Supplementary Information [file 41467_2020_17011_MOESM1_ESM.pdf]

SUPPLEMENTARY INFORMATION

Oncolytic virus-derived type I interferon restricts CAR T cell therapy

Evgin et al.

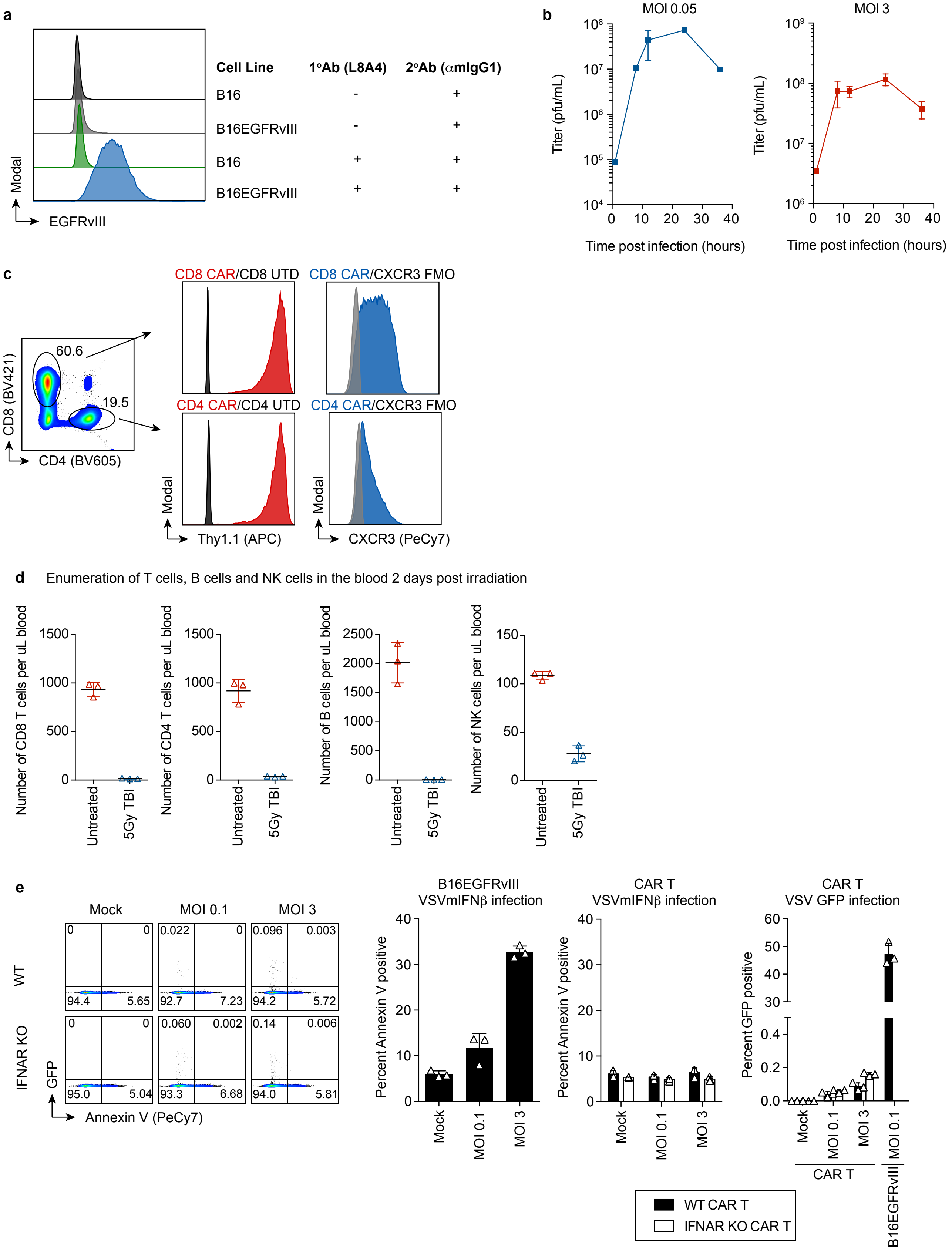
a
Cell Line
1°Ab (L8A4)
2°Ab (αmIgG1)
B16 - +
B16EGFRvIII - +
B16 + +
B16EGFRvIII + +
Modal
EGFRvIII
b
MOI 0.05
MOI 3
Titer (pfu/mL)
Time post infection (hours)
c
60.6
19.5
CD8 (BV421)
CD4 (BV605)
CD8 CAR/CD8 UTD
CD8 CAR/CXCR3 FMO
CD4 CAR/CD4 UTD
CD4 CAR/CXCR3 FMO
Modal
Thy1.1 (APC)
CXCR3 (PeCy7)
d
Enumeration of T cells, B cells and NK cells in the blood 2 days post irradiation
Number of CD8 T cells per uL blood
Number of CD4 T cells per uL blood
Number of B cells per uL blood
Number of NK cells per uL blood
Untreated
5Gy TBI
e
Mock
MOI 0.1
MOI 3
WT
IFNAR KO
GFP
Annexin V (PeCy7)
0
0
94.4
5.65
0.022
0
92.7
7.23
0.096
0.003
94.2
5.72
0
0
95.0
5.04
0.060
0.002
93.3
6.68
0.14
0.006
94.0
5.81
B16EGFRvIII VSVmIFNβ infection
CAR T VSVmIFNβ infection
CAR T VSV GFP infection
Percent Annexin V positive
Percent GFP positive
CAR T
B16EGFRvIII
WT CAR T
IFNAR KO CAR T

Supplementary Figure 1. Phenotypic characterization of the model and the permissivity of relevant cell types to VSV (**A**) B16 cells were engineered using a pBABE vector to express the murine analog of EGFRvIII(*47*) modified by the deletion of 500 amino acids from the intracellular domain of the protein. EGFRvIII is recognized using the L8A4 clone and a secondary anti-mouse IgG1. (**B**) Single step and multi-step growth curves were conducted in B16EGFRvIII tumor cells left and right panels, respectively. Viral supernatants were collected at the indicated time points and titered on BHK cells. Mean titers ± SD for technical duplicates are shown (**C**) Murine splenocytes were stimulated with ConA (2.5 ug/mL) and IL2 (50 U/mL) for two days and transduced with the third generation EGFRvIII CAR IRES Thy1.1 vector. On day 5 of culture, the transduction efficiency was monitored by Thy1.1 staining on CD8 and CD4 cells compared to untransduced (UTD) cells. The expression of the CXCR3 chemokine receptor was also measured on day 5 and compared to the fluorescence minus one (FMO) control. (**D**) Lymphodepletion validation two days after 5 Gy TBI. B cells, T cells and NK cells were quantified from submandibular vein bleeds. Mean ± SD for n=3 mice/group is shown. (**E**) B16EGFRvIII and day 5 CAR T cells generated from WT C57/Bl6 or transgenic IFNAR1 KO mice were infected with either VSV GFP or VSVmIFNβ at and MOI of 0.1 or 3 for 24h. Representative expression of GFP is plotted against Annexin V expression (left panel). Annexin V staining in tumor cells or CAR T cells infected with VSVmIFNβ at an MOI 0.1 or 3 for 24h is shown (middle panels). Comparison of the permissivity of CAR T cells and B16EGFRvIII to the expression of GFP from VSV (right panel). Mean ± SD for N=3 technical replicates is shown. Source data are provided in the Source Data File.

Endpoint tumor staining with DAPI; EGFRvIII

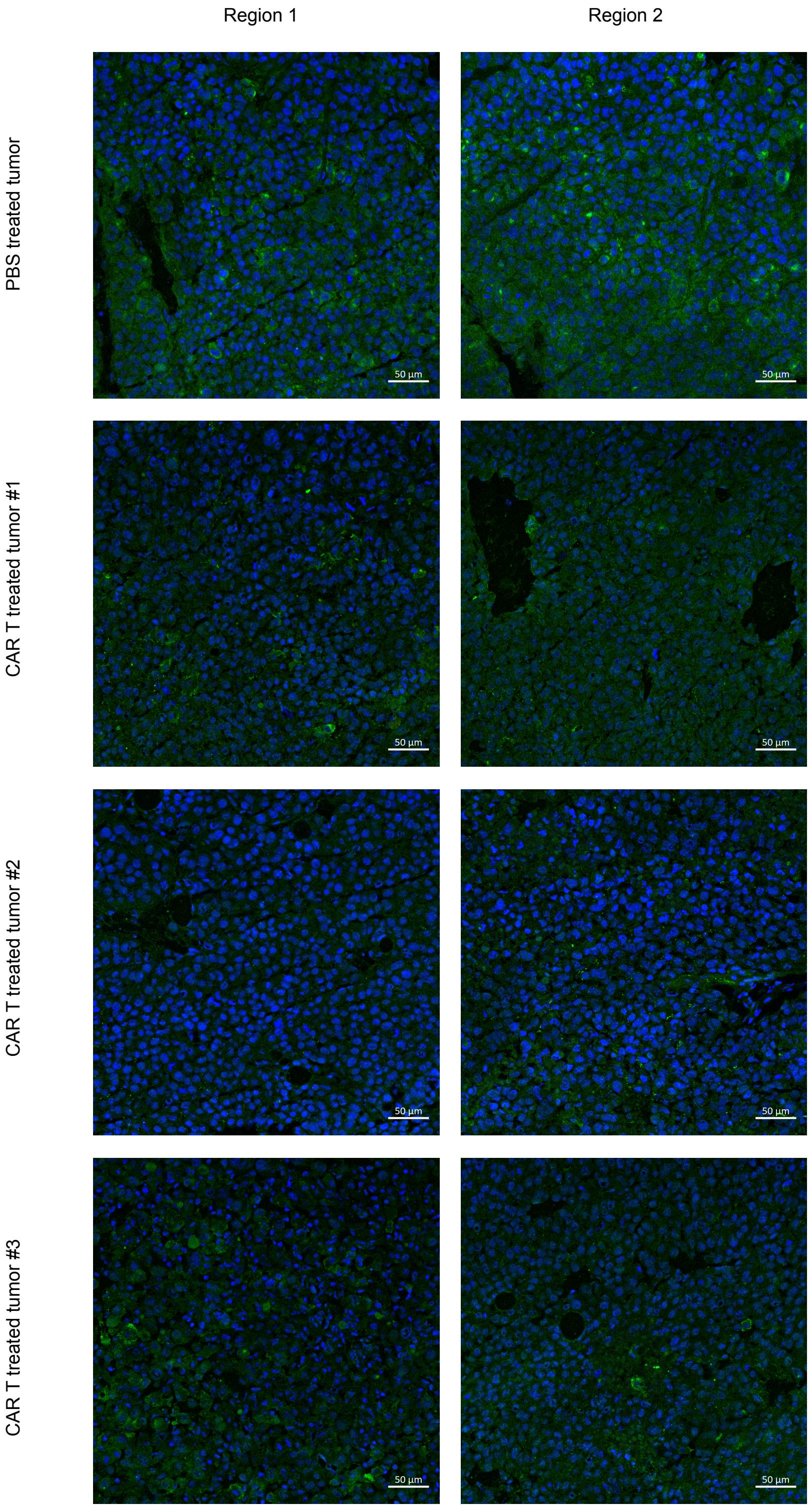

Supplementary Figure 2. EGFRvIII antigen expression on B16EGFRvIII tumors *in vivo*. Mice bearing B16EGFRvIII tumors were irradiated on day 6 (5Gy TBI) and received 100 uL PBS IV (top row) or 10^7 EGFRvIII specific CAR T cells (bottom three rows) on day 7. When tumors reached endpoint criteria, tumors were frozen and sections were stained with the L4A8 anti-EGFRvIII antibody and anti-mouse IgG1-AF488. Staining was performed once and images selected were representative of the entire section. Scale bar represents 50 μm.

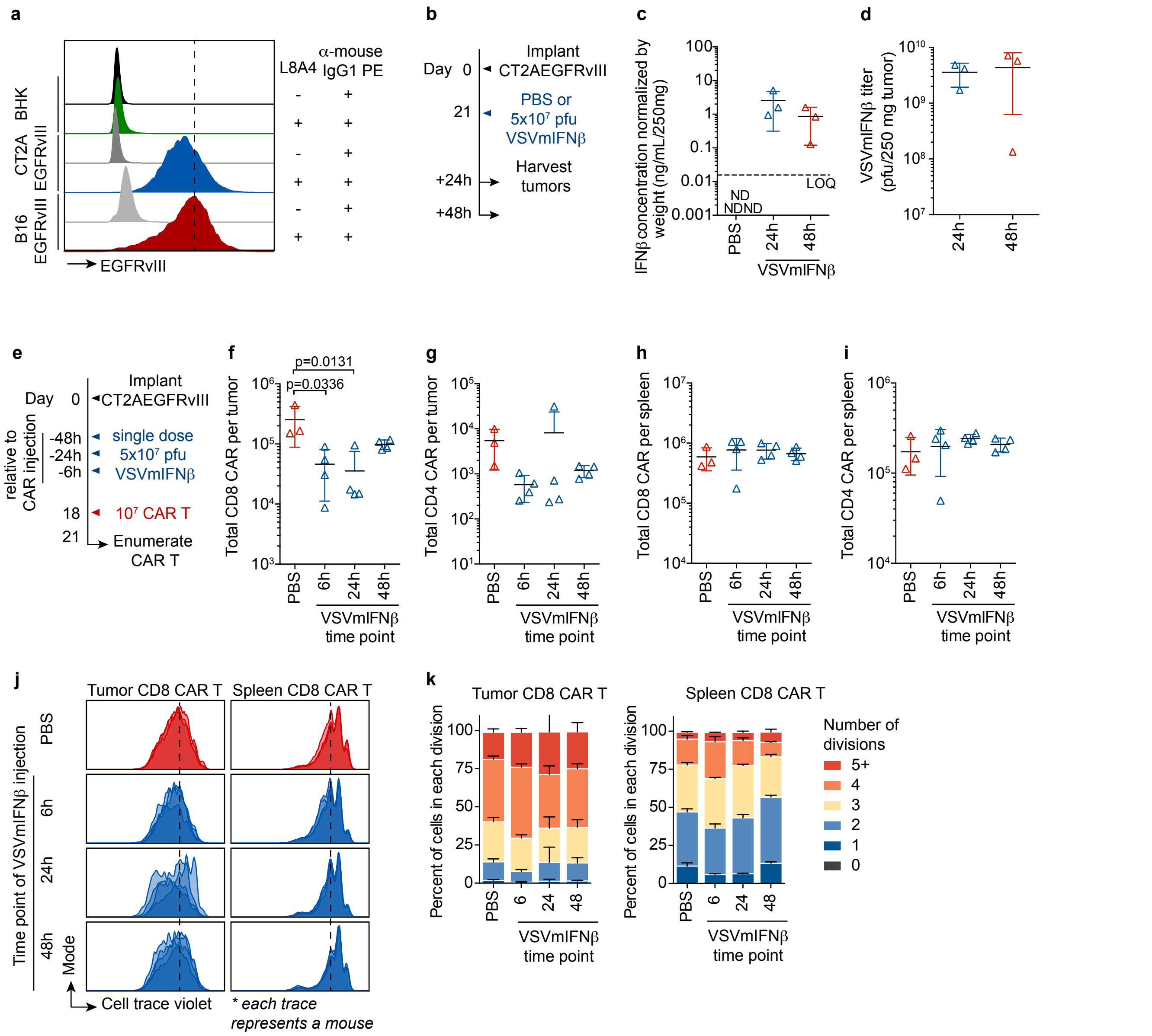

Supplementary Figure 3. CT2AEGFRvIII tumor model of VSVmIFNβ-associated attrition. (A) EGFRvIII expression on CT2AEGFRvIII and B16EGFRvIII compared to control BHK cells, as measured by staining with the L8A4 clone and anti-mouse IgG1 PE. **(B)** Mice bearing CT2AEGFRvIII tumors were injected intratumorally with PBS or $5x10^7$ pfu VSVmIFNβ. 24 or 48 hours later, mice were euthanized and tumors harvested. Mean ±SD for n=3 mice/group and each symbol represents a mouse. Tumor homogenates were quantified for levels of IFNβ by ELISA **(C)** or infectious virus **(D)**. **(E)** Mice bearing CT2AEGFRvIII tumors were injected intratumorally with PBS or $5x10^7$ pfu VSVmIFNβ at various time points prior to adoptive transfer of 10^7 EGFRvIII CAR T cells. **(F-I)** CD8 and CD4 CAR T cells were enumerated in the tumor and spleen 72 hours post adoptive transfer. Mean ±SD for n=3 or 4 mice/group and each symbol represents a mouse. **(J)** Cell proliferation was monitored by cell trace violet dilution. The cell trace violet dilution for all mice per group is overlaid. **(K)**The mean percent of cells in each division ±SD for n=3 or 4 mice/group is shown. P-values were determined using a one-way ANOVA with a Tukey multiple comparisons post-test in **F**. Source data are provided in the Source Data File.

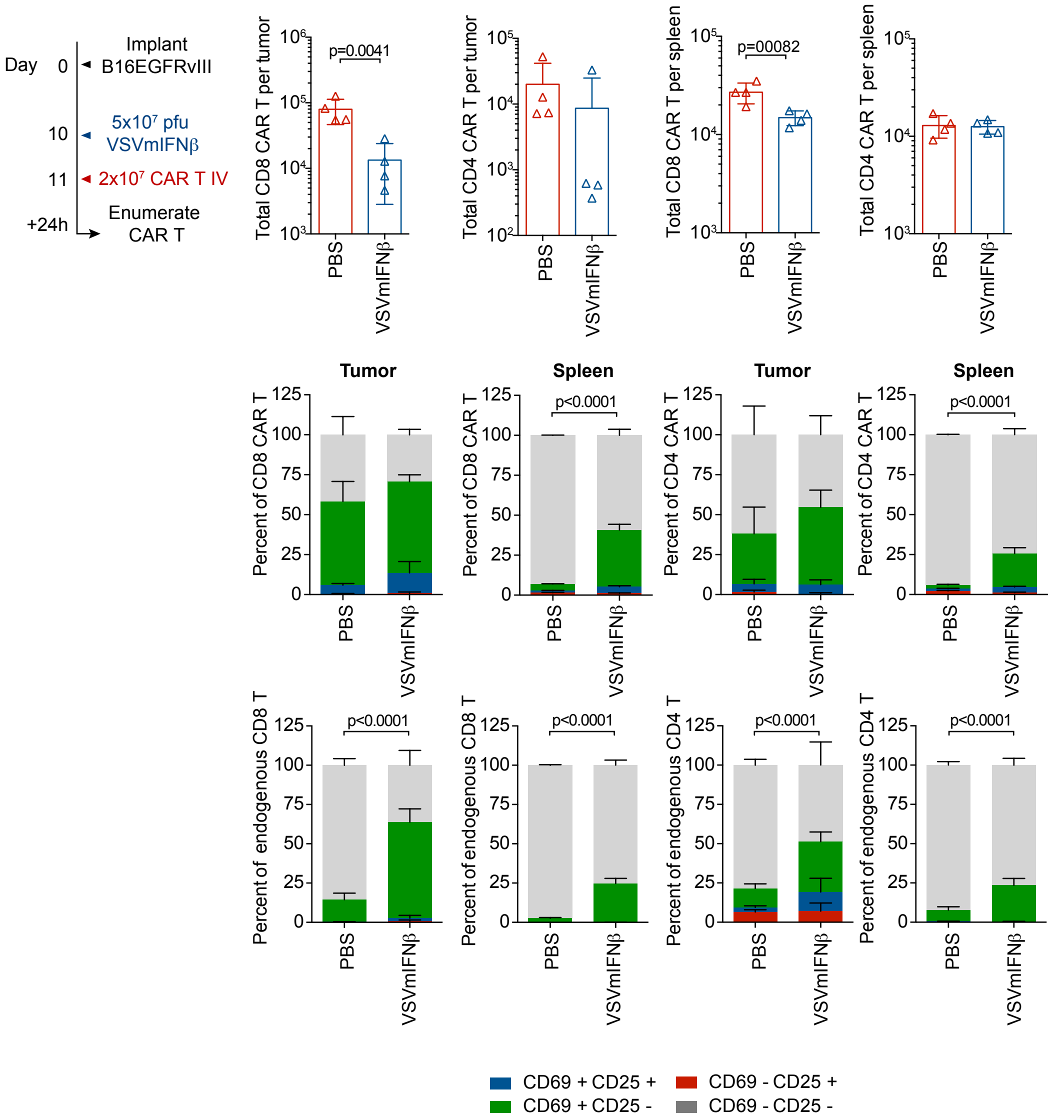

Supplementary Figure 4. CAR T cell activation *in vivo* following VSVmIFNβ. Mice bearing B16EGFRvIII subcutaneous tumors were treated intratumorally with PBS or 5x10^{7} VSVmIFNβ 24 hours prior to adoptive transfer of 2x10^{7} EGFRvIII CAR T cells. CAR T cells were enumerated in the tumor and spleen and mean values ±SD for n=4 mice/group are shown (top row). Activation markers on CAR T cells and endogenous T cell populations were quantified and mean values ±SD for n=4 mice/group are shown (bottom panels). Activation maker statistical comparisons are indicated for the CD69^{-}CD25^{-} population in each treatment group. P-values were determined using an unpaired two-tailed t test on log transformed data (CAR T enumeration) or a two-way ANOVA with a Sidak's multiple comparisons post-test (activation markers). Source data are provided in the Source Data File.

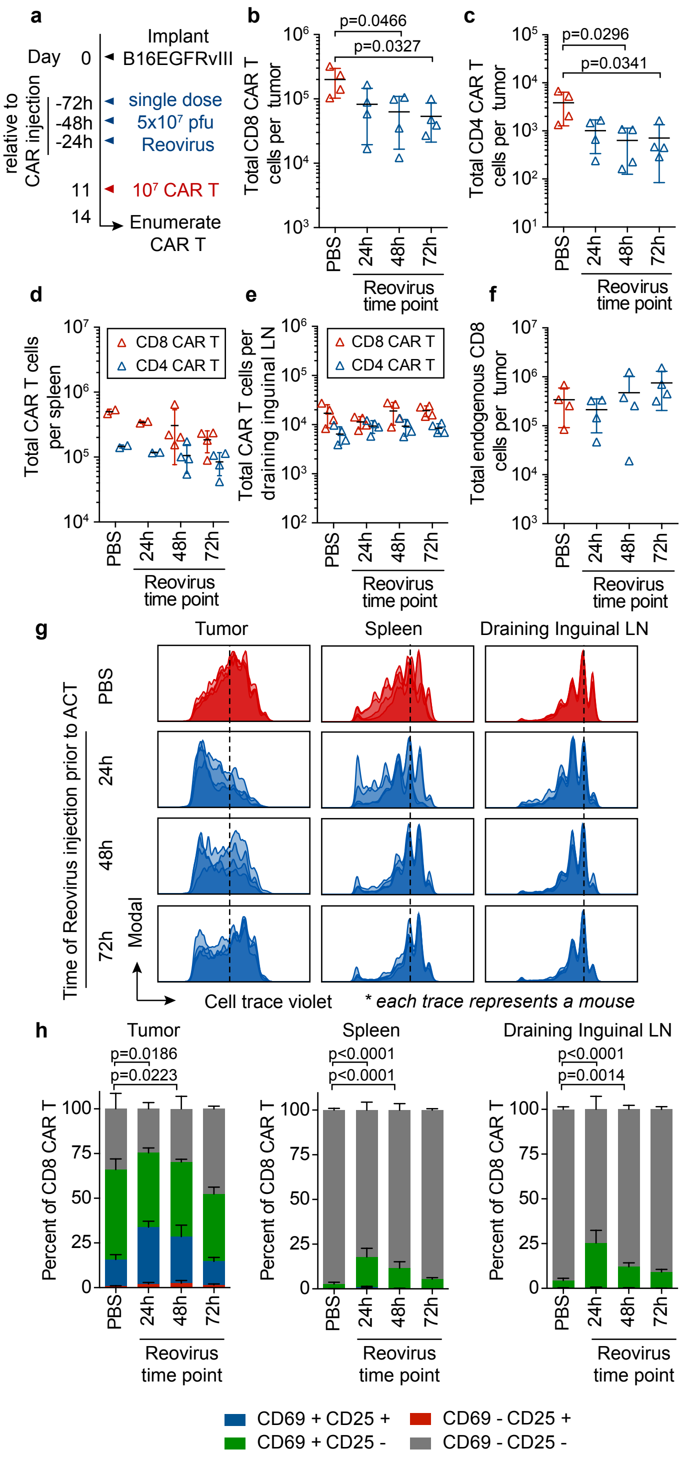

Supplementary Figure 5. Oncolytic reovirus promotes CAR T cell attrition, proliferation and activation. (A) Mice received a single intratumoral injection of PBS or 5×10^7 pfu of Reovirus 24, 48, or 72 hours prior to adoptive transfer of 1×10^7 EGFRvIII CAR T cells on day 11. CD8 **(B)** and CD4 **(C)** Thy1.1$^+$ CAR T cells were enumerated in the tumor three days after adoptive transfer and represented as mean ± SD for n=4/group. **(D,E)** Total CD8 and CD4 CAR T cells were enumerated in the spleen and draining inguinal lymph node respectively and represented as mean ±SD for n=4/group except in the spleens for the PBS and 24h timepoint groups where n=2/group. **(F)** Endogenous Thy1.1$^-$ CD8 cells were quantified in the tumor and represented as mean ±SD for n=4/group. **(G)** CAR T cell proliferation was monitored by cell trace violet dilution in the tumor, spleen and draining inguinal lymph node. The cell trace violet dilution for all four mice per group is overlaid. **(H)** Activation as measured by CD69 and CD25 in the tumor, spleen and lymph node is represented as mean ± SD for n=4/group. Activation maker statistical comparisons are indicated for the CD69$^+$ CD25$^-$ population in each treatment group. P-values were determined using a one-way ANOVA with a Tukey multiple comparisons post-test **(B,C)** or a two-way ANOVA with a Tukey multiple comparisons post-test **(H)**. Source data are provided in the Source Data File.

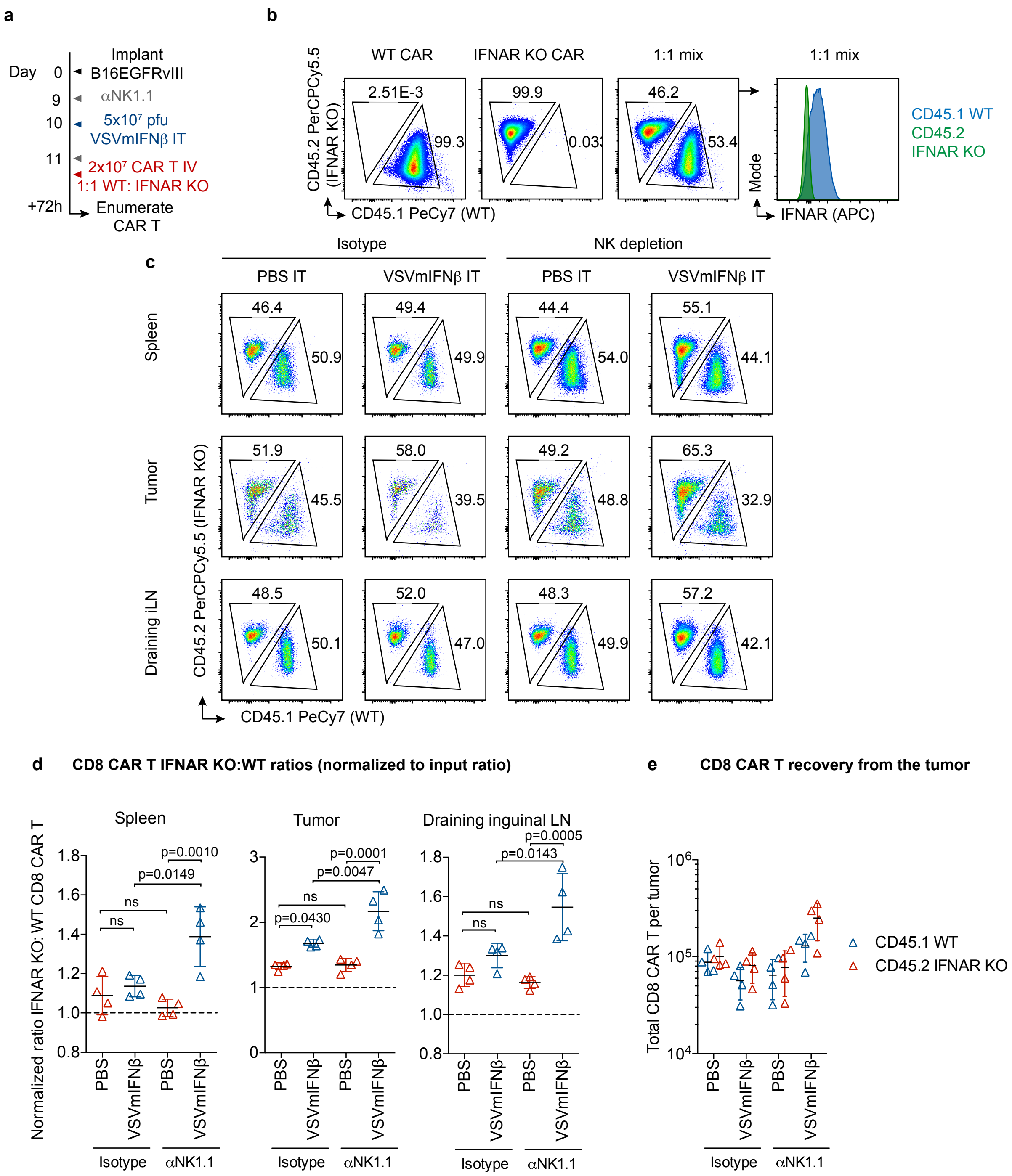

Supplementary Figure 6. IFNAR KO CAR T cells outcompete WT CAR T cells following VSVmIFNβ in the context of NK cell depletion. (A) Mice bearing B16EGFRvIII subcutaneous tumors received two intraperitoneal doses of 300 μg of anti-NK1.1 antibody or control mouse IgG on days 9 and 11. On day 10 mice were treated intratumorally with PBS or 5x10^7 pfu of VSVmIFNβ. On day 11, mice received a 1:1 mixture of WT and IFNAR KO CAR T cells (1x10^7 each) bearing the congenic markers CD45.1 and CD45.2, respectively **(B)**. 72 hours after adoptive transfer, CAR T cells were quantified in the spleen, tumor, and tumor draining inguinal lymph node. Representative flow plots are shown in **(C)** and the ratios of CD8 IFNAR KO: WT cells (CD45.2/CD45.1) normalized to the input ratio are represented as mean ±SD for n=4/group in **(D)**. **(E)** Total CD8 CAR T cell counts recovered from the tumor are represented as mean ±SD for n=4/group. Data is representative of two independent experiments. P-values were determined using a one-way ANOVA with a Tukey multiple comparisons post-test **(D)**. Statistical significance set at p<0.05, ns >0.05. Source data are provided in the Source Data File.

Supplementary Figure 8.

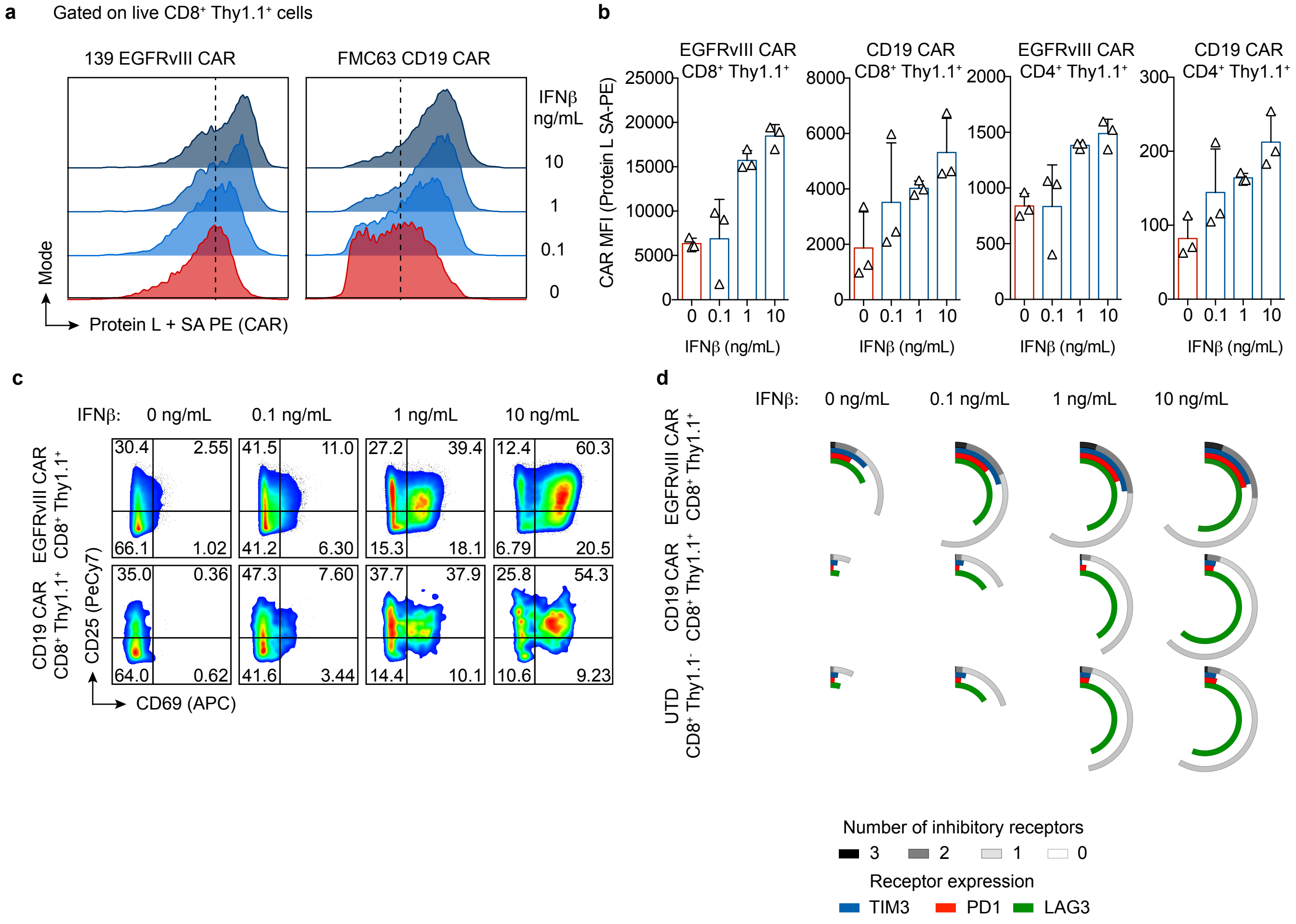

Supplementary Figure 8. CD19 CAR T cells treated with IFNβ in vitro. EGFRvIII specific CAR T cells (with the scFv 139) and CD19 specific CAR T cells (with the scFv FMC63) were cultured in IL2 in the absence or presence of recombinant murine IFNβ and CAR expression on the surface was measured using Biotin-Protein L and streptavidin- PE (Protein L + SA PE). Representative staining is shown in **(A)** and the mean ± SD of n=3 technical replicates is quantified in **(B)**. **(C)** Representative expression of the activation markers CD25 and CD69 were shown for CAR T cells cultured as in panel A. **(D)** Inhibitory receptor expression (PD1, LAG3, TIM3) quantified on CD8 CAR T or UTD cells cultured as in panel A and represented as the mean expression for n=3 technical replicates.

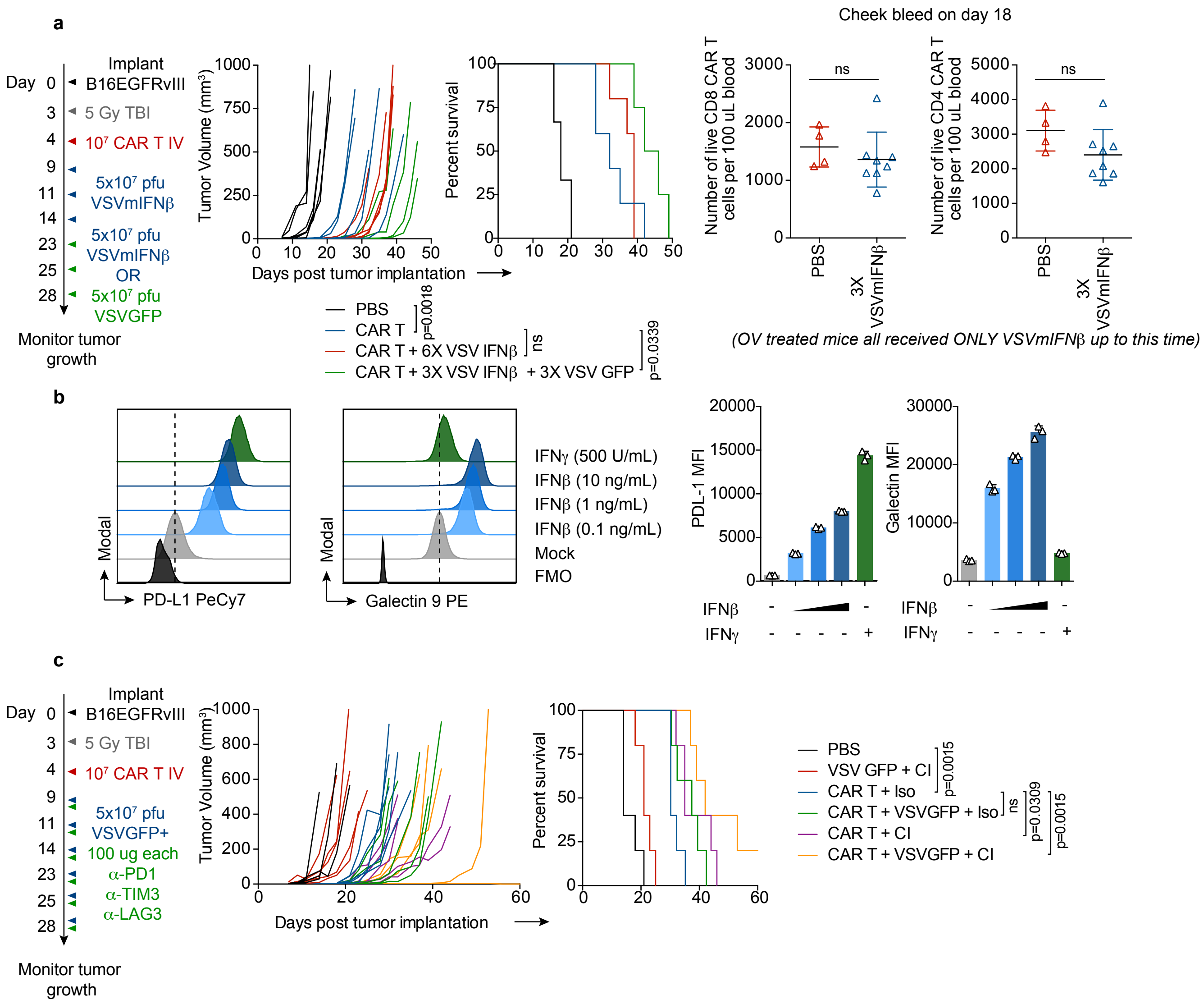

Supplementary Figure 9. Alternative scheduling combination CAR T cells with VSVGFP. (A) Mice bearing B16EGFRvIII tumors were given a lymphodepleting dose of radiation (5 Gy TBI) on day 3 and were treated with $1x10^7$ EGFRvIII CAR T cells on day 4. Mice were intratumorally treated with $5x10^7$ pfu VSVmIFNβ or PBS on days 9,11 and 14. Select groups received 3 additional doses of either $5x10^7$ pfu VSVmIFNβ or VSV GFP on days 23,25,28. Overall survival and tumor growth is shown for n=6 mice/group (middle panels). CAR T cells in the blood were quantified on day 18 (right panel) and represented as mean ±SD for n=4 (PBS) or n=8 (VSVmIFNβ) groups. **(B)** B16EGFRvIII cells were treated with recombinant IFNβ or IFNγ at the indicated doses for 24h. Representative surface PD-L1 and intracellular Galectin 9 staining (left panels) as well as the means ±SD of n=3 technical replicates (right panels) is shown. **(C)** Mice bearing B16EGFRvIII tumors were given a lymphodepleting dose of radiation (5 Gy TBI) on day 3 were treated with $1x10^7$ EGFRvIII CAR T cells on day 4. Mice were intratumorally treated with $5x10^7$ pfu VSVGFP as well as a cocktail of checkpoint inhibitors (CI) consisting of 100 μg each of anti-PD1, anti-TIM3 and anti-LAG 3, or 300 μg of control IgG. Overall survival and tumor growth is shown for n=5/group. P-values were determined using a Log-rank Mantel-Cox test (**A,C**) or an unpaired two-tailed t test (**A**, CAR enumeration in blood). Statistical significance set at $p<0.05$. ns >0.05. Source data are provided in the Source Data File.

Sample gating strategy for murine CAR T cells (Thy1.1+ve)

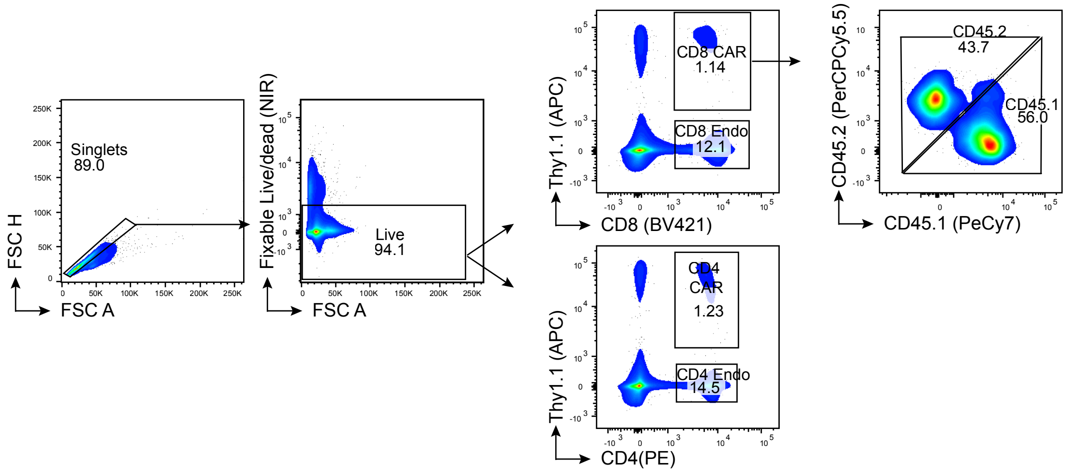

Sample gating strategy for human CAR T cells (PepvIII tetramer +ve)

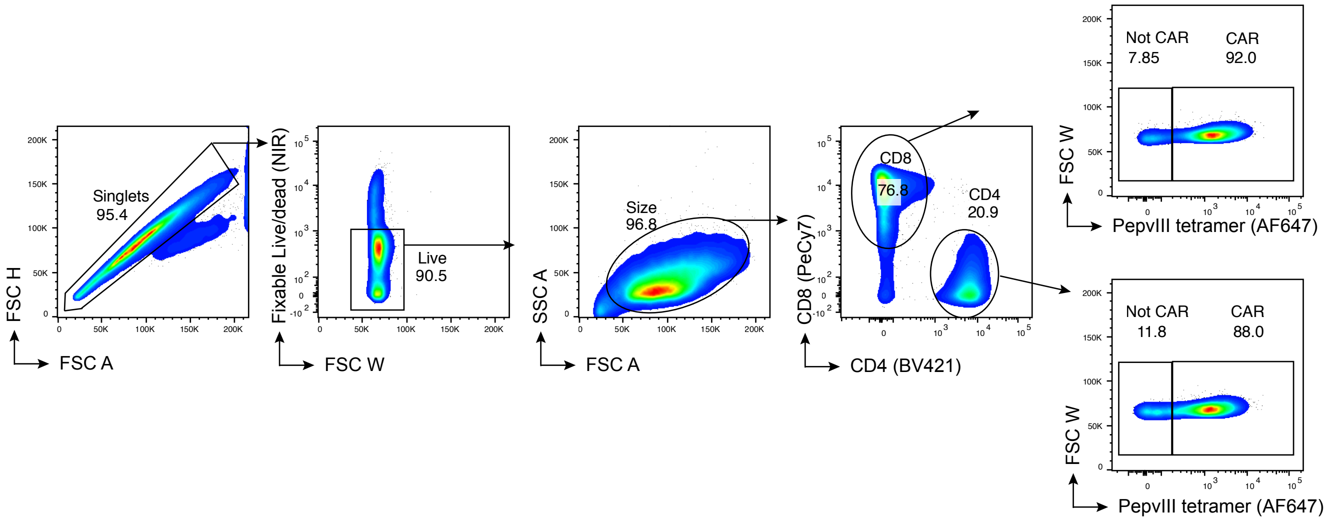

Supplementary Figure 10. Sample gating strategy for CAR T cells. Cell debris were excluded using the FSC-A and SSC-A parameters. Doublets were excluded using the FSC-A and FSC-H parameters. Live cells were FLD-NIR negative. Mouse CAR T cells were identified as $CD8^+$ or $CD4^+$ $Thy1.1^+$. Congenic markers CD45.1 and CD45.2 were used to distinguish IFNAR KO and WT donors. Human CAR T cells were identified as $CD8^+$ or $CD4^+$ PepvIII $tetramer^+$. Further gating strategies depend on the particular experimental setup where positive/negative parameters were gated based on FMOs.
