# Supplementary material for: Oncolytic virus-derived type I interferon restricts CAR T cell therapy
Source: Nat Commun. 2020 Jun 24;11:3187. doi: 10.1038/s41467-020-17011-z (PMC7314766; doi:10.1038/s41467-020-17011-z)
Supplement: Supplementary file 3 — Reporting Summary [file 41467_2020_17011_MOESM3_ESM.pdf]

nature research

Corresponding author(s): Richard Vile

Last updated by author(s): May 18, 2020

Reporting Summary

Nature Research wishes to improve the reproducibility of the work that we publish. This form provides structure for consistency and transparency in reporting. For further information on Nature Research policies, see our Editorial Policies and the Editorial Policy Checklist.

Statistics

For all statistical analyses, confirm that the following items are present in the figure legend, table legend, main text, or Methods section.

n/a	Confirmed	
☐	☒	The exact sample size (*n*) for each experimental group/condition, given as a discrete number and unit of measurement
☐	☒	A statement on whether measurements were taken from distinct samples or whether the same sample was measured repeatedly
☐	☒	The statistical test(s) used AND whether they are one- or two-sided<br>*Only common tests should be described solely by name; describe more complex techniques in the Methods section.*
☐	☒	A description of all covariates tested
☐	☒	A description of any assumptions or corrections, such as tests of normality and adjustment for multiple comparisons
☐	☒	A full description of the statistical parameters including central tendency (e.g. means) or other basic estimates (e.g. regression coefficient) AND variation (e.g. standard deviation) or associated estimates of uncertainty (e.g. confidence intervals)
☐	☒	For null hypothesis testing, the test statistic (e.g. *F*, *t*, *r*) with confidence intervals, effect sizes, degrees of freedom and *P* value noted<br>*Give P values as exact values whenever suitable.*
☒	☐	For Bayesian analysis, information on the choice of priors and Markov chain Monte Carlo settings
☒	☐	For hierarchical and complex designs, identification of the appropriate level for tests and full reporting of outcomes
☒	☐	Estimates of effect sizes (e.g. Cohen's *d*, Pearson's *r*), indicating how they were calculated

Our web collection on statistics for biologists contains articles on many of the points above.

Software and code

Policy information about availability of computer code

Data collection	FACSDiva v8.0 software (on the FACSCanto X SORP), Everest™ v2.0 software (on the ZE5 Cell Analyzer), Zeiss's Zen software Black edition v8.0 (LSM 780 inverted confocal microscope).
Data analysis	Adobe Illustrator CC 2017, Flowjo 10.5, Graphpad Prism 6, Microsoft Excel 2011, Zeiss's Zen software Black edition v8.0, Legendplex software 7.1.0.0

For manuscripts utilizing custom algorithms or software that are central to the research but not yet described in published literature, software must be made available to editors and reviewers. We strongly encourage code deposition in a community repository (e.g. GitHub). See the Nature Research guidelines for submitting code & software for further information.

Data

Policy information about availability of data

All manuscripts must include a data availability statement. This statement should provide the following information, where applicable:

- Accession codes, unique identifiers, or web links for publicly available datasets
- A list of figures that have associated raw data
- A description of any restrictions on data availability

All data generated during this study are available from the corresponding author upon reasonable request. The source data underlying all main and supplemental figures are provided as a Source Data file.
Animals were randomized to treatment groups using the GraphPad QuickCalcs online tool (https://www.graphpad.com/quickcalcs/randMenu/).

Field-specific reporting

Please select the one below that is the best fit for your research. If you are not sure, read the appropriate sections before making your selection.

- [x] Life sciences
- [ ] Behavioural & social sciences
- [ ] Ecological, evolutionary & environmental sciences

For a reference copy of the document with all sections, see nature.com/documents/nr-reporting-summary-flat.pdf

Life sciences study design

All studies must disclose on these points even when the disclosure is negative.

Sample size	Although statistical methods were not used to predetermine sample size, sample sizes were chosen on the basis of estimates from pilot experiments and previously published results. Similar numbers of animals were used in PMID: 24352643. For in vitro mouse T cell studies, a minimum of triplicates was chosen to allow for calculation of statistics. For in vitro human T cell studies, cells were collected from 3 independent donors and performed in duplicate.
Data exclusions	T cell enumeration was performed in tumors weighing > 50 mg. Enumeration of CAR T cells in spleens with poor overall viability from Fig 2F,G and Supplemental Fig 5D were not included in the analysis. These exclusion criteria were pre-established. No in vitro data was excluded.
Replication	In vitro experiments were typically run with triplicate technical replicates for mouse studies or technical duplicates for human studies in cells from three independent donors and were reproduced at least twice. Most in vivo studies were performed twice with similar results. Some in vivo studies were performed once, although control treatment groups were repeated across many experiments and demonstrate reproducible effects.
Randomization	All animals included in the study were randomized to treatment groups following tumor implantation using the GraphPad QuickCalcs online tool (https://www.graphpad.com/quickcalcs/randMenu/). For in vitro studies, samples were allocated to identical cell culture systems and there is no reason to believe that spatial organization influenced experimental outcomes.
Blinding	The investigators were not blinded to the allocation of groups during experiments or subsequently during the analysis. Fully blinded experiments were not possible due to personnel availability to accommodate such situations.

Reporting for specific materials, systems and methods

We require information from authors about some types of materials, experimental systems and methods used in many studies. Here, indicate whether each material, system or method listed is relevant to your study. If you are not sure if a list item applies to your research, read the appropriate section before selecting a response.

Materials & experimental systems

n/a	Involved in the study	
[ ]	[x]	Antibodies
[ ]	[x]	Eukaryotic cell lines
[x]	[ ]	Palaeontology and archaeology
[ ]	[x]	Animals and other organisms
[ ]	[x]	Human research participants
[x]	[ ]	Clinical data
[x]	[ ]	Dual use research of concern

Methods

n/a	Involved in the study	
[x]	[ ]	ChIP-seq
[ ]	[x]	Flow cytometry
[x]	[ ]	MRI-based neuroimaging

Antibodies

Antibodies used	Mouse flow cytometry antibodies: CD3 (Biolegend # 100236 clone 145-2C11, dilution 1:500), CD8a (Biolegend #100738/100747, clone 53-6.7, dilution 1:1000), CD8b (Biolegend # 140410, clone 53-5.8, dilution 1:500), CD4 (Biolegend #100451 clone GK1.5), Thy1.1 (Biolegend # 202524, clone OX-7, dilution 1:1000; or eBioscience # 11-0900-85, cloneH1S51, dilution 1:500), CD19 (biolegend # 115537, clone 6D5, dilution 1:500), NK1.1 (Biolegend 108706/7, clone PK136, dilution 1:100), CXCR3 (Biolegend #126515, clone CXCR3-173, dilution 1:100), IFNAR1 (Biolegend # 127314, clone MAR1-5A3, dilution 1:100), PD1 (Biolegend # 109110, clone RMP1-30, dilution 1:200), LAG3 (Biolegend # 125212, clone C9B7W, dilution 1:200), TIM3 (Biolegend #119704, clone RMT3-23, dilution 1:200), CD25 (Biolegend # 102016, clone PC61 or BD # 553866, clone PC61, dilutions both 1:200), CD69 (Biolegend # 104513, clone H1.2F3, dilution 1:200), CD45 (Biolegend # 103114, clone 30-F11, dilution 1:500), CD45.1 (Biolegend # 110728/9, clone A20, dilution 1:250), CD45.2 (Biolegend # 109828, clone 104, dilution 1:250), PD-L1 (Biolegend # 124314, clone 10F.9G2, dilution 1:200), Fas (Biolegend # 152604/152612, clone SA367H8, dilution 1:200), cleaved caspase (BD Pharmingen # 51-68655X, clone C92-605). Transduction efficiency was determined using the Thy1.1 marker as well as biotinylated protein L (GenScript, dilution 1:100) and streptavidin–phycoerythrin, SA-PE (BD Biosciences # 554061, dilution 1:100) or streptavidin– allophycocyanin, SA-APC (Invitrogen# 1932748)<br><br>Human flow cytometry antibodies:CD4 (Biolegend # 317433, clone OKT4, dilution 1:250), CD8 (Biolegend # 300914, clone HIT8a,

dilution 1:250), PD1 (Biolegend # 329923, clone EH12.2H7, dilution 1:50), Tim3 (Biolegend # 345015, clone F38-2E2, dilution 1:50), and Lag3 (Biolegend # 369319, clone 11C3C65, dilution 1:50) and cleaved caspase 3 (R& D systems # IC835G, clone 269518, dilution 1:100)

EGFRvIII expression: anti-human EGFRvIII antibody clone L8A4 (Absolute Antibody # Ab00184-1.1, dilution 1:100) and anti-mouse IgG1 (clone RMG1-1 Biolegend # 406607 or 406625).

Human CAR T cells preparation: anti-CD3 OKT3 antibody (Biolegend # 317326)
In vivo experiments: anti-TIM3 (clone RMT-23; BioXCell# BE0115), anti-PD1 (clone RMP1-14; BioXCell # BE0146), and anti-mLAG3 (clone C9B7W; BioXCell # BE0174), control rat IgG (Jackson ImmunoResearch # 012-000-003), NK1.1 (clone PK136; BioXCell # BE0036) or 300 ug of control mouse IgG (JacksonImmunoResearch # 015-000-003).

Validation

All antibodies used for flow cytometry were validated by the manufacturer directly. In our laboratory, antibody-specific staining was compared to FMO samples.

Anti-mouse CD3 was validated using C57BL/6 mouse splenocytes https://www.biolegend.com/en-us/products/apc-anti-mouse-cd3-antibody-8055
Anti-mouse CD8 alpha was validated using C57BL/6 mouse splenocytes https://www.biolegend.com/en-us/products/brilliant-violet-421-anti-mouse-cd8a-antibody-7138
Anti-mouse CD8 beta was validated using C57BL/6 mouse splenocytes
https://www.biolegend.com/en-us/products/apc-anti-mouse-cd8b-2-antibody-6872
Anti-mouse CD4 was validated using C57BL/6 mouse splenocytes https://www.biolegend.com/en-us/products/brilliant-violet-605-anti-mouse-cd4-antibody-10708
Ant-Thy1.1 was validated using LOU rat thymocytes https://www.biolegend.com/en-us/products/pe-anti-rat-cd90-mouse-cd90-1-thy-1-1-antibody-5620 or rat splenocytes https://www.thermofisher.com/antibody/product/CD90-1-Thy-1-1-Antibody-clone-HIS51-Monoclonal/11-0900-81
Anti-mouse CD19 was validated using C57BL/6 mouse splenocytes https://www.biolegend.com/en-us/products/brilliant-violet-421-anti-mouse-cd19-antibody-7160
Anti-mouse NK1.1 was validated using C57BL/6 mouse splenocytes https://www.biolegend.com/en-us/products/fitc-anti-mouse-nk-1-1-antibody-429
Anti-mouse CXCR3 was validated using C57BL/6 mouse splenocytes
https://www.biolegend.com/en-us/products/pe-cyanine7-anti-mouse-cd183-cxcr3-antibody-6169
Anti-mouse IFNAR1 was validated using C57BL/6 mouse splenocytes https://www.biolegend.com/en-us/products/apc-anti-mouse-ifnar-1-antibody-4785
Anti-mouse PD-1 was validated using Con A-stimulated (day-3) Balb/c mouse splenocytes https://www.biolegend.com/en-us/products/pe-cyanine7-anti-mouse-cd279-pd-1-antibody-3612
Anti-LAG-3 was validated using Con A-stimulated splenocytes
https://www.biolegend.com/en-us/products/percp-cyanine5-5-anti-mouse-cd223-lag-3-antibody-8141
Anti-TIM-3 was validated using Mouse Tim-3 transfected cells
https://www.biolegend.com/en-us/products/pe-anti-mouse-cd366-tim-3-antibody-2657
Anti-mouse CD25 was validated using Con A-stimulated (2 days) BALB/c mouse splenocytes
https://www.biolegend.com/en-us/products/pe-cyanine7-anti-mouse-cd25-antibody-1929 or BALB/c bone marrow leukocytes https://www.bdbiosciences.com/eu/applications/research/t-cell-immunology/regulatory-t-cells/surface-markers/mouse/pe-rat-anti-mouse-cd25-pc61/p/553866
Anti-mouse CD69 was validated using Con A-stimulated C57BL/6 mouse splenocytes (2 days)
https://www.biolegend.com/en-us/products/apc-anti-mouse-cd69-antibody-3169
Anti-mouse CD45 was validated using C57BL/6 mouse splenocytes https://www.biolegend.com/en-us/products/pe-cyanine7-anti-mouse-cd45-antibody-1903
Anti-mouse CD45.1 was validated using SJL mouse splenocytes
https://www.biolegend.com/en-us/products/percp-cyanine5-5-anti-mouse-cd45-1-antibody-4269
Anti-mouse CD45.2 was validated using C57BL/6 mouse splenocytes
https://www.biolegend.com/en-us/products/percp-cyanine5-5-anti-mouse-cd452-antibody-4271
Anti-mouse PDL-1 was validated using C57BL/6 mouse splenocytes https://www.biolegend.com/en-us/products/pe-cyanine7-anti-mouse-cd274-b7-h1--pd-l1-antibody-6721
Anti-mouse Fas was validated using C57BL/6 thymocytes
https://www.biolegend.com/en-us/products/apc-anti-mouse-cd95-fas-antibody-13906
Anti-cleaved caspase was validated using capothectin treated cells https://www.bdbiosciences.com/eu/reagents/research/cell-based-assays/apoptosis/pe-rabbit-anti--active-caspase-3-c92-605/p/550821
Anti-human CD4 was validated using human peripheral blood lymphocytes
https://www.biolegend.com/en-us/products/brilliant-violet-421-anti-human-cd4-antibody-7775
Anti-human CD8 was validated using human peripheral blood lymphocytes https://www.biolegend.com/en-us/products/pe-cyanine7-anti-human-cd8a-antibody-1916
Anti-human PD-1 was validated using human peripheral blood lymphocytes
https://www.biolegend.com/en-us/products/brilliant-violet-605-anti-human-cd279-pd-1-antibody-7671
Anti-human TIM-3 was validated using PHA-stimulated (3-day) human peripheral blood lymphocytes https://www.biolegend.com/en-us/products/percp-cyanine5-5-anti-human-cd366-tim-3-antibody-8438
Anti-human LAG-3 was validated using CD3/CD28/IL-2 stimulated (three days) peripheral blood mononuclear cells (PBMCs) https://www.biolegend.com/en-us/products/brilliant-violet-711-anti-human-cd223-lag-3-antibody-14878
Anti-cleaved caspase was validated using Jurkat human acute T cell leukemia cells treated with 3 µM Staurosporine for 3 hours
https://www.rndsystems.com/products/human-mouse-cleaved-caspase-3-alexa-fluor-488-conjugated-antibody-269518_ic835g

Anti-EGFRvIII was validated using A431 cells https://absoluteantibody.com/product/anti-egfrviii-l8a4/Ab00184-1.4_Mouse_IgG1/
Anti-mouse IgG1was validated using Human peripheral blood lymphocytes were stained with purified anti-human CD3 (clone UCHT1, mouse IgG1 isotype) followed by anti-mouse IgG1 (clone RMG1-1). https://www.biolegend.com/en-us/products/pe-anti-mouse-igg1-6494

Eukaryotic cell lines

Policy information about cell lines

Cell line source(s)	B16 EGFRvIII was generated from B16.F1 originally obtained from ATCC; BHK (ATCC); L929 (ATCC); CT2AEGFRvIII (John Sampson, Duke University), 293T (Larry Pease, Mayo Clinic, originally from ATCC), PG13-139-CD8-CD28BBZ-F10 (Steven Rosenberg NIH).
Authentication	EGFRvIII expression was validated by flow cytometry for the B16EGFRvIII and CT2AEGFRvIII cell lines using the anti-EGFRvIII antibody clone L8A4. We have not performed genetic testing of the cell lines.
Mycoplasma contamination	Cell lines were routinely tested for mycoplasma and found to be negative.
Commonly misidentified lines (See ICLAC register)	No commonly misidentified cell lines were used in the study.

Animals and other organisms

Policy information about studies involving animals; ARRIVE guidelines recommended for reporting animal research

Laboratory animals	Female C57BL/6 (stock 000664) (CD45.2) and B6.SJL-Ptprca Pepcb/BoyJ (stock 002014) (CD45.1) mice were obtained from The Jackson Laboratory and female B6.129S2-Ifnar1tm1Agt/Mmjax (stock 32045-JAX) (IFNAR1 KO; CD45.2) mice were obtained from MMRC JAX. All mice were obtained at 6–8 weeks of age and maintained in a specific pathogen-free BSL2 biohazard facility. Pmel mice (originally obtained from The Jackson Laboratory (stock 005023); Thy1.1, CD45.2) were bred at the Mayo Clinic, and splenocytes from female mice were harvested between 8-14 weeks of age for adoptive transfer experiments. Mice were exposed to a 12:12 h light-dark cycle with unrestricted access to water and food. The ambient temperature was restricted to 68 to 79 degrees F and the room humidity ranged from 30-70%.
Wild animals	The study does not involve wild animals.
Field-collected samples	The study does not involve field collected samples.
Ethics oversight	All animal studies were conducted in accordance with and approved by the Institutional Animal Care and Use Committee at Mayo Clinic.

Note that full information on the approval of the study protocol must also be provided in the manuscript.

Human research participants

Policy information about studies involving human research participants

Population characteristics	PBMCs were collected from three healthy human donors (40 year old female, 33 year old male, 56 year old male) that donated blood to the Mayo Clinic Blood Donor Center.
Recruitment	Peripheral blood mononuclear cells (PBMCs) from healthy donor apheresis cones were obtained through the Mayo Clinic Blood Donor Center.
Ethics oversight	The use of research cones was approved by the Division of Transfusion Medicine Research Committee at Mayo Clinic and determined to be IRB exempt.

Note that full information on the approval of the study protocol must also be provided in the manuscript.

Flow Cytometry

Plots

Confirm that:

- [x] The axis labels state the marker and fluorochrome used (e.g. CD4-FITC).
- [x] The axis scales are clearly visible. Include numbers along axes only for bottom left plot of group (a 'group' is an analysis of identical markers).
- [x] All plots are contour plots with outliers or pseudocolor plots.
- [x] A numerical value for number of cells or percentage (with statistics) is provided.

Methodology

Sample preparation	Flow cytometry was performed on cultured cells or freshly explanted spleens, blood, tumors, livers, lungs, lymph nodes or bone marrow. Tumors were weighed and treated with Liberase TL (Roche) and DNAse I (Sigma) for 30-45 min at 37C. 30 mg of tumor or other tissue was stained and run on the flow cytometer. 100 uL of blood collected by submandibular vein bleed was subjected to red blood cell lysis and stained. With the exception of cells stained with Annexin V which were resuspended in Annexin V binding buffer (Biolegend), samples were fixed in 4% formaldehyde.
Instrument	FACSCanto X SORP), ZE5 Cell Analyzer
Software	FACSDiva v8.0 software (on the FACSCanto X SORP), Everest™ v2.0 software (on the ZE5 Cell Analyzer)
Cell population abundance	Sorting was not performed.
Gating strategy	Cell debris were excluded using the FSC-A and SSC-A parameters. Doublets were excluded using the FSC-A and FSC-H parameters. Live cells were FLD-Zombie negative. Further gating strategies depend on the particular experimental setup. Positive/negative parameters were gated based on FMOs.

☒ Tick this box to confirm that a figure exemplifying the gating strategy is provided in the Supplementary Information.
